# Probing Viral Dark Matter: Comparative Genomics of Atypical Bacillus Phage YungSlug

**DOI:** 10.3390/v17091267

**Published:** 2025-09-19

**Authors:** Allison A. Johnson, Andrew Hale, Amine Sehnouni, Zainab Gbadamosi, Bret M. Boyd

**Affiliations:** School of Life Sciences and Sustainability, Virginia Commonwealth University, Richmond, VA 23284, USA

**Keywords:** bacteriophage, *Bacillus*, *Spounavirinae*, virus diversity

## Abstract

*Bacillus* phage YungSlug is a novel phage with a genome that has limited homology to known viruses. To better understand this unique phage, we searched for close relatives of YungSlug using traditional comparative genomics approaches and a broad search of a large protein database. YungSlug shares only 9% genome alignment with Bacillus phage Nachito, its closest relative, and less than 1% with others in its subfamily. A search for homologs in the NCBI nr database was dominated by low percent identity homologs from viral, bacteria, and environmental sources, returning matches for only 50% of the predicted proteins in YungSlug’s genome. Additionally, a set of 21 conserved proteins was identified that may define a core gene set for the *Spounavirinae* subfamily of *Herelleviridae*. These findings highlight the diversity of phages infecting *Bacillus* and highlight gaps in our knowledge of the *Bacillus*-infecting phage community.

## 1. Introduction

The HHMI SEA PHAGES (Science Education Alliance-Phage Hunters Advancing Genomics and Evolutionary Science) program [1] seeks to document the diversity of phages infecting select bacterial species. While many newly isolated phages cluster with previously described viruses based on homology, evolutionarily distinct phages continue to emerge. It is unclear if these novel phages represent viruses that rarely infect those bacterial species or if detection and classification methods preferentially select phages in certain clusters. To help address this problem, we further examined a genomically well-characterized phage, YungSlug [2], a phage that shares little homology with known phages, using broad database searches.

The SEA PHAGES program has deposited a combined 5123 sequenced phage genomes in NCBI databases (phagesdb.org [3], bacillus.phagesdsb.org (accessed on 15 July 2025)), adding to a rapidly growing collection. As the availability of phage genome sequences increases, newly isolated and sequenced phage genomes are interpreted within the context of this larger dataset. Here, we expand analysis on a recently isolated phage infecting *Bacillus thuringiensis* [2], examining the genome within the context of curated *Bacillus*-infecting phage genomes and more broadly through a search of viral, bacterial, and environmental sequences.

There are an estimated 10^31^ bacteriophages present on Earth, establishing phages as the most abundant and genetically diverse entities on our planet [4,5,6]. Interactions between phage and bacteria are potentially the most frequent biological interaction, with the results of these interactions regulating host population densities, shaping host evolution, altering global carbon cycles, and impacting human health [5,7,8,9,10]. However, the specific nature in which phage genomes evolve can confound our ability to understand the phylogenetic origins of many phage isolates.

Studies of phages that infect a single host genus have revealed a staggering sequence level variation within homologous regions, as well as novel combinations of protein-encoding genes [11,12,13,14,15,16]. Phage genome evolution is shaped by horizontal gene exchange, likely leading to the acquisition of both homologous and non-homologous genes or groups of genes within and between phylogenetic clusters [11,12,14] in both lytic and temperate phages. However, horizontal exchange appears to have had a larger impact on the evolution of temperate phage, which integrate their genome into the host genome, than on lytic phage, which are obligate host-killing parasites that do not integrate into the host genome [17]. For example, the lytic *Bacillus*-infecting phages, phiAGATE, SPO1, Shanette, JL, CP-51, Bastille [17] have comparatively lower rates of horizontal gene exchange than temperate phage parasitizing the same host species [6,14,18,19,20].

Bacterial species in the genus *Bacillus* are ubiquitous in natural ecosystems, with some species being pathogenic [21,22], and have been used in industrial biotechnology applications [23]. Studies of phages that infect *Bacillus* provide insights into the ecology and evolution of bacteriophages impacting a large medically and economically important group of bacteria. Because of their significance, *Bacillus* phages have been characterized through many individual studies, including the isolation of phages that may be useful for identification and biocontrol of pathogenic hosts [24,25,26,27]. Larger-scale comparative studies have supported results observed with other host genera, showing enormous genetic diversity as well as little genome similarity between phage clusters [12,14,28,29,30]. They have also revealed a large number of functionally uncharacterized protein-coding genes [12,14,28,29,30], and clear genome boundaries between lytic and prophage sequences [30]. *Bacillus* phage can specifically impact unique properties of their hosts, such as sporulation [31,32,33], the formation of bacterial cell walls and biofilms [30,31], and regulation of host defenses [34]. Yet with the exception of a single comparative genomics study analyzing phage 0305phi8-36 [35], there has been relatively little focus on examining phage diversity from the perspective of “under-sampled” *Bacillus* phage.

At several SEA PHAGES hosting institutions, *B. thuringiensis* has been used to isolate phage from the environment, adding to the >419 unique *Bacillus* phage genomes that have been deposited in NCBI databases, with many genomes closely matching previously sequenced phage genomes. As a result, most of the *B. thurinigiensis* phage form phylogenetically distinct clusters, with multiple members in each cluster, using traditional clustering methods [12,14,36]. However, in a few instances, studies have isolated phages, which do not cluster with other phages (specifically those known to infect the same host species) [35]. Here we examine one such phage, “YungSlug”, a lytic phage infecting *B. thuringiensis*, that was isolated by students enrolled in a SEA PHAGES course in 2018 at Virginia Commonwealth University from a water sample collected from the James River near Richmond, VA [2]. The initial genome annotation [2] revealed YungSlug’s 150,011 bp genome lacked significant alignment to any other genome and deserved further study. Of YungSlug’s 227 predicted proteins, function was predicted for 36%, while 50% of proteins lack homologs in GenBank. In this study, we searched genome and protein databases for close relatives of YungSlug using traditional approaches as well as through a broad search of a large protein database. Specifically, we ask if any homologs are shared between YungSlug and other phages, bacteria, eukaryotes, and metagenomic assemblies. From this, we characterize the genetic relationship of an atypical *Bacillus* phage YungSlug to 12 other sequenced and characterized *Bacillus* phages from the *Spounavirinae* and *Bastillevirinae* subfamilies within *Herelleviridae*.

## 2. Materials and Methods

Genomes included in this study: the YungSlug genome was compared to the genome sequences of 12 phages that infect five different *Bacillus* species hosts (Table 1). Genomes were selected to represent commonly sampled ‘populations’ (NotTheCreek, Bastille, Juglone), as well as those that are more unique and lack homology depth in GenBank. These phages represent the ICTV taxa *Spounavirinae* (n = 8) and *Bastillevirinae* (n = 4) subfamilies within Family *Herelleviridae* [36]. Phage 0305phi8-36 remains characterized in the Class Caudoviricetes.

Phamerator genome database: The comparative genomics tool Phamerator (V1.1) [37] was used to store and visualize a custom database containing 143 Bacillus phage genomes. Phamerator organizes proteins from these phages into phamilies, based on 32.5% sequence identity to another protein, and was also used to visualize genome maps.

Protein content analysis: Phage similarity based on the presence and absence of protein phamilies was visualized as a network phylogeny using Splitstree (V4.17.0) [38]. A custom Python program was written to calculate pairwise gene content similarity (GCS) [39]. Briefly, the protein phamily presence and absence table from Phamerator was used to generate pairwise counts of the number of shared proteins. These counts were averaged to determine the pairwise GCS score of two different genomes. A heat map of GCS scores was visualized using the R programming language.

Dot plot analysis: Regions of identity between whole genome and proteome sequences were visualized using the software Gepard (V1.4) [40]. A text file of concatenated sequences was compared against itself. Word size was set to 15 for nucleotide comparisons and 5 for amino acid comparisons.

Genome-wide examination of relatedness: In order to observe the relatedness of our chosen phages to other organisms, we queried each of the phages’ protein sequences against the NCBI non-redundant protein database (NCBI nr, downloaded 27 October 2022) using BLASTP. In order to optimize computing resources, these protein sequences were split into three to six separate files, and a batch job was submitted for each. After the jobs were completed, the BLASTP outputs were concatenated together for each of the phages. The concatenated final BLASTP output files were filtered to remove self-hits as well as query-target below 30% sequence similarity. The subject IDs remaining after filtering were used to obtain the subject NCBI taxonomy string and classification using Batch Entrez. R was used to visualize a genome-wide dot plot of the best bacterial and virus hits, as well as create histogram profiles of all the hits binned by percent identity.

## 3. Results

Bacillus phage YungSlug was discovered in 2018 in an undergraduate phage discovery course. Phage plaques were 2 mm in diameter and clear. While we were unable to obtain a transmission microscopy image to verify phage particle morphology, the presence of tail tube and tail sheath genes, as well as synteny to other genomes, supports a prediction of myoviridae morphology. The genome characteristics of YungSlug were initially reported in 2021 [2] MT416612.2, with a note that YungSlug may be an atypical phage.

The *Bacillus*-infecting phage Nachito (OP380492) shared the greatest sequence similarity to YungSlug, with an overall genome alignment coverage of 9% of the YungSlug genome and an average percent identity of 80% within homologous regions. All other phages examined share <1% of the YungSlug genome.

**Figure 1:**
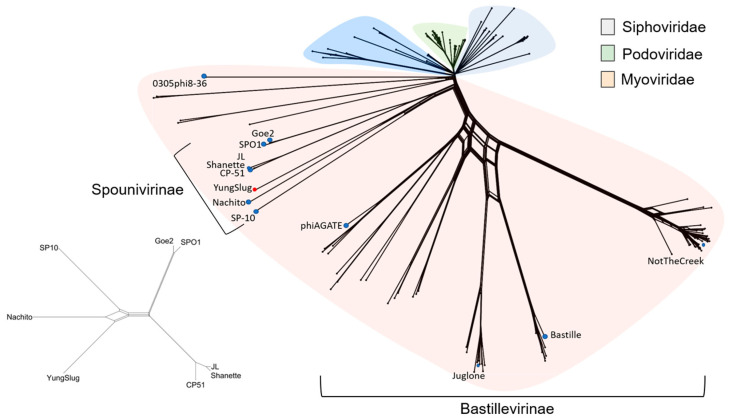
Phylogenetic networks showing the relationship of YungSlug and other *Bacillus*-infecting phages, where patristic distance was inferred from the shared functional homologs, or phams, shared between each phage genome. The network is color-coded by older physical morphology classifications, and brackets show current ICTV subfamily groupings. The inset panel shows an isolated network for select *Spounivirinae* phages. Blue and red dots indicate phage genomes selected for this study.

Because homologs were not identified by BLASTN, we turned to protein-based methods to understand relationships to other sequenced phages. Protein families (herein phams) were identified using Phamerator (amino acid sequences sharing ≥ 32.5% of identical sites were clustered in Phamerator as functional homologs [37]). Genome-wide pham content was used to construct a network phylogeny of *Bacillus*-infecting phage (Figure 1). The resulting network places YungSlug within the *Spounivirinae* subfamily. Similar grouping is observed using the ViPTree server [41]. The network also indicates a high frequency of closely related phages in the subfamily *Bastillevirinae* were frequently isolated, indicated by phage clusters separated by short distances in the network. In comparison, closely related phages were less frequently sampled from the *Spounivirinae*, with phages often separated by longer network edges. The closest relative of YungSlug in this network is Nachito, but these phages are separated by a comparatively long distance in the network. This network was used to select a comparison set of phages across the ICTV *Spounivirinae* and *Bastillevirinae* subfamilies of *Herelleviridae* for this study (Figure 1, blue and red dots).

**Figure 2:**
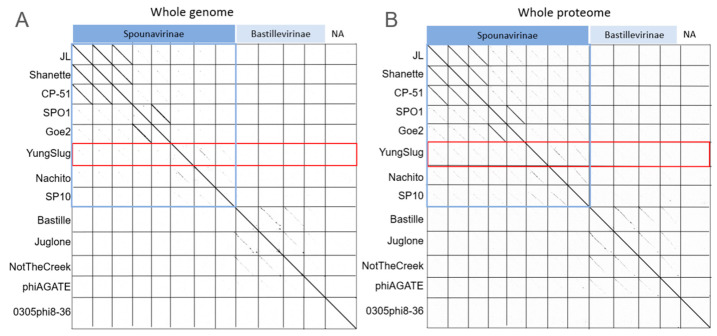
Syntenic blocks shared between *Bacillus*-infecting phage, based on the phage’s genome (**A**) and proteome (**B**). Plot constructed using Gepard^1^, with either a 15 bp nucleotide or 5 amino acid word size. Phage names are on the left of the plots, and labels above the plots indicate ICTV taxonomy subfamily groupings.

YungSlug’s genome and proteome sequences were compared to 12 selected phage genomes and proteomes (Table 1), these being representative of the genomic diversity of *Bacillus*-infecting phage. The selected phage belongs to the subfamilies *Spounavirinae* and *Bastillevininae* (Family *Herelleviridae*) [36], as well as one unclassified *Caudoviricetes*. Analysis of genome identity by dot plot found diffuse genomic sequence conservation between YungSlug and Nachito, while YungSlug shares little to no homologous regions with other *Spounavirinae* or *Bastillevirinae* phages examined (Figure 2A). When using the proteome to identify regions with conserved amino acid sequences, we see an increase in the detection of candidate homologous regions within *Spounavirinae* (Figure 2B), indicating greater conservation of the protein sequences compared to the genome sequences.

**Table 1:** Genome characteristics of the *Bacillus* phage in this study.

Phage	GenBank Accession	Host	Genome Length (bp)	GC%	# ORFs	Publication
Subfamily: *Spounavirinae*						
CP-51	KF554508.2	*B. cereus*	138,658	40.9	219	[42,43]
JL	KC595512.2	*B. cereus*	137,918	40.8	222	[44]
Shanette	KC595513.2	*B. cereus*	138,877	40.8	223	[44]
SP-10	NC_019487.1	*B. subtilis*	143,986	40.5	236	[45]
SP01	FJ230960.1	*B. subtilis*	132,562	40	209	[46]
Goe2	KY368639.1	*B. subtilis*	146,141	40.3	230	[47]
YungSlug	MT416612.2	*B. thuringiensis kurstaki*	150,011	37.6	227	[2]
Nachito	OP380492	*Bacillus* sp. ET1 lab isolate	148,928	38.5	238	Unpublished
Subfamily: *Bastillevirinae*						
Bastille	JF966203.1	*B. cereus* HER1399 ^a^	153,962	38.1	273	[42,48]
Juglone	KU737345.1	*B. thuringiensis kurstaki*	164,227	37.8	293	[49]
NotTheCreek	KU737351.1	*B. thuringiensis kurstaki*	161,929	38.7	296	[49]
phiAGATE	JX238501.3	*B. pumilus*	149,844	41	204	[50]
Class: *Caudoviricetes*						
0305phi8-36	EF583821.1	*B. thuringiensis*	218,948	41.8	247	[35,51]

^a^ GenBank record lists *B. cereus* HER1399 as host, Ackerman’s publication says *B. thuringiensis* is the host.

**Figure 3:**
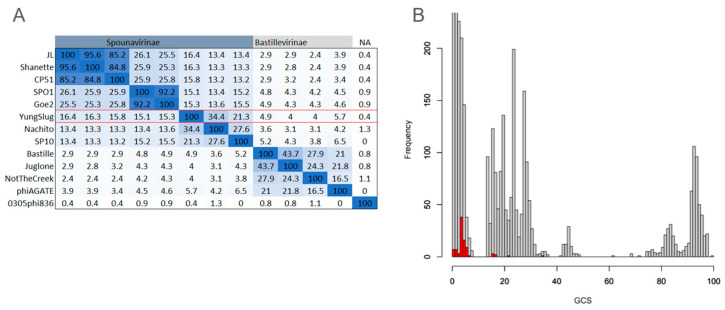
Pairwise gene content similarity (GCS) comparison for 13 *Bacillus*-infecting phages (**A**) and heatmap plot showing the distribution of GCS values calculated by pairwise comparisons of 143 *Bacillus*-infecting phages (**B**). In (**B**), values derived from comparisons of YungSlug and other phages are highlighted in red, following the removal of duplicate and self-matches.

To quantify the overall functional similarity between YungSlug and other *Bacillus*-infecting phages, we calculated the GCS [39] score through the identification of shared protein phams. The highest GCS score was obtained from the comparison of YungSlug and Nachito, with a score of 34% (Figure 3A), derived from a set of 79 proteins present in both genomes. Comparisons of YungSlug to other phages yielded scores less than 34%. When examining all pairwise GCS scores of *Bacillus*-infecting phages (not just comparisons involving YungSlug), we find that YungSlug falls on the lower end of GCS score distribution (GCS score range = 1.3–100%; n = 143, Figure 3B red bars). For example, within *Spounavirinae* and *Bastillevirinae,* phages share between 13–100% and 15–100% of their protein families, respectively, with comparisons of phages in the same phylogenetic cluster yielding a GCS score > 80%.

**Figure 4:**
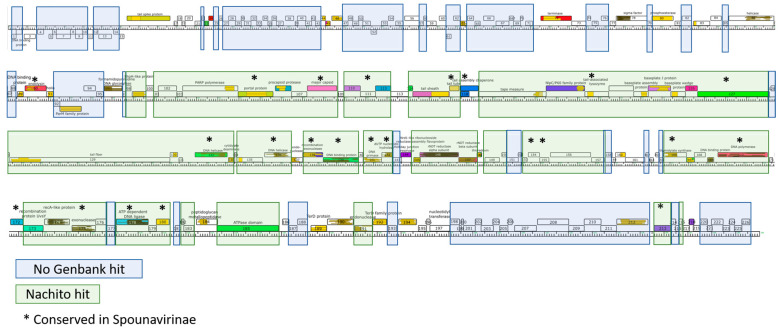
Genome map of YungSlug. Proteins are indicated by rectangles above or below the genome ruler. Green shaded boxes indicate protein regions with homology to *Bacillus* phage Nachito. Blue shading indicates proteins with no BLASTP hit to the GenBank nr database. Asterisks indicate 21 protein functions that are conserved in the subfamily *Spounavirinae*-classified phages included in this study.

While 34% of proteins were shared between YungSlug and Nachito, extensive regions of the YungSlug genome contain proteins with no obvious homology to any protein in the NCBI protein nr database (blue shaded regions, Figure 4). Fifteen proteins were identified as conserved (>30% amino acid similarity) in the *Spounavirinae* genomes in our study (Supplemental Table S1; Figure 4), including two phage structural proteins (tail-associated lysozyme and major capsid protein), eight DNA-modifying enzymes (DNA repair exonuclease SbcCD ATPase subunit, helicase, thymidylate synthase, DNA primase, exonuclease, dUTP pyrophosphatase, DNA polymerase and ATP-dependent DNA ligase), two putative RNA polymerase sigma factors and three hypothetical proteins that are functionally uncharacterized. An additional six proteins were identified as conserved more broadly, across *Bastillevirinae* as well as *Spounavirinae*: these include terminase large subunit, portal protein, DNA repair exonuclease SbcCD nuclease subunit, tail tube protein, DNA helicase, and a hypothetical protein of an unknown function (Supplemental Table S1; Figure 4, see asterisks). In total, this set of 21 homologous proteins in *Spounavirinae* subfamily genomes suggests a core set of functions focused on structural and DNA-modifying enzymes.

**Figure 5:**
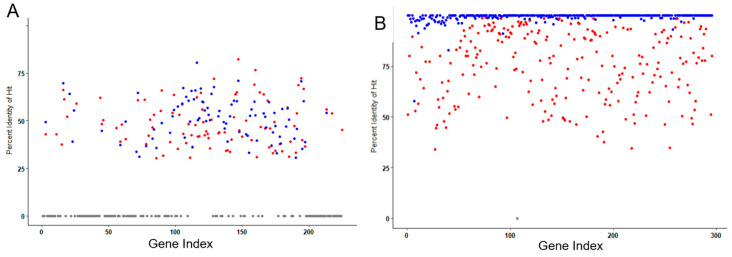
Comparison of the best BLASTP hits to YungSlug proteins originating from phage and bacterial genomes. Each point represents the best BLASTP hit in the NCBI non-redundant protein database for each protein in the YungSlug (**A**) and NotTheCreek (**B**) phage genomes. Best hits originating from phage genomes are colored in red, those from bacterial genomes are colored in blue. Gray dots indicate that a best hit, with sequence similarity ≥ 30% was not found.

To examine protein sequence homology relationships more broadly, all predicted amino acid sequences in the YungSlug genome were compared to the proteins in the NCBI nr database using BLASTP, using a search database of candidate homologs in bacteria, viruses, and environmental samples (accepting BLASTP hits with 30–100% identity). Of the 227 predicted amino acid sequences in the YungSlug genome, 50% (113 proteins) did not return a significant BLAST hit (Figure 5A, gray dots). The greatest sequence similarity came from viral hits (blue dots, largely hits to Nachito), with the maximum sequence identity of 82%. For comparative purposes, the search was replicated for seven other *Bacillus*-infecting phages. Here we illustrate search returns when proteins from phage NotTheCreek served as the queries (Figure 5B). Of the 296 predicted proteins encoded in NotTheCreek’s genome, only one sequence failed to return a BLAST hit. Nearly all the amino acid sequences returned a close viral hit, with percent identity for each being >90% (Figure 5B, blue dots). Likewise, numerous hits were returned from bacterial genomes, ranging from 30 to 98% identity (Figure 5B, red dots).

**Figure 6:**
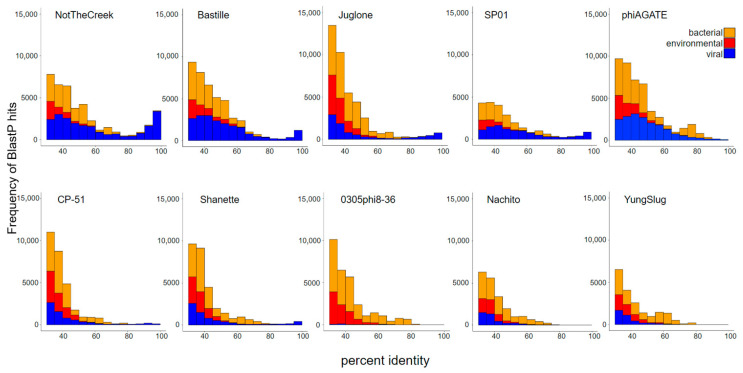
Frequency of BLASTP hits in the NCBI non-redundant protein database for ten distinct *Bacillus*-infecting phages, binned by percent identity. Stacked frequency bars are color-coded by bacteria (yellow), environmental (red), and viral (blue) origin.

Results for the other phage examined lie between the results obtained for YungSlug and NotTheCreek (Figure 6). The profile of hits for each phage is dominated by low percent identity homologs from viral, bacteria, and environmental sources. The frequency and percent identity of virus-derived hits for more frequently sampled phages like NotTheCreek are higher than those characteristics for infrequently sampled phages like 0305phi8-36, Nachito, and YungSlug. Despite the enormous contributions of viral sequences from environmental metagenomic studies [6], high-quality hits from these sources were not observed when using these phage proteomes as queries.

Finally, with YungSlug as a query, we examined the total number of BLASTP hits originating from a single genome, allowing us to identify viral or bacterial genomes that may share multiple homologs with YungSlug. We found that Nachito yielded more hits than any other individual genome identified in the BLAST search, as expected, and that other genomes lacked hits, such as gene repeats.

## 4. Discussion

A rich dataset of genomes of *Bacillus*-infecting phage has enabled work to improve understanding of the diversity and evolution of this group of viruses [12,28,29,30]. Barylski (2020) found that “SPO1” related viruses formed a distinct network cluster and established the family *Herelleviridae*, with five potential subfamilies [36]. They reported nucleotide sequence similarities are ‘virtually undetectable’ above the rank of genus, but protein sequences remained considerably similar. This study focused on the analysis of 12 phage genomes from two of those subfamilies, *Bastillevirinae*, *Spounavirinae*, and the unclassified 0305phi8-36. In this study, we expand on these methods by incorporating a search of the NCBI nr database and rigorously sorting the results to identify viral, bacterial and metagenomic homologous sequences. We conclude that YungSlug represents a novel phage cluster; its genome contains 113 predicted proteins that are novel; and we define a core set of conserved proteins in the subfamily *Spounivirinae*.

We characterized the novel lytic phage YungSlug, which has limited genetic relationships to other viruses and represents the sole described member of an evolutionarily distinct phage cluster in our analysis (see [17] for discussion of evolutionarily distinct phage clusters). Our analysis, which included other *Bacillus*-infecting phages, also indicated that other clusters were only represented by one or two phage isolates (generally *Spounavirinae*), while other phages grouped into clusters with several isolates (generally *Bastillevirinae*). This begs the question, why are some lytic phage clusters rarely sampled, while others are sampled more often, when sampling phage infecting one or a few closely related bacterial species? We propose the following, non-mutually exclusive hypotheses:

(1) Isolation methods favor specific phage types. Methods used by the SEA PHAGES program for the detection and isolation of phage primarily from soil rely on a homogenous environment with a single host genotype [52] that may fail to replicate important environmental features or host-phage interactions [53,54,55,56]. Likewise, phages may have characteristics that make them difficult to detect, even if able to successfully infect host cells under culture conditions [57,58]. The use of novel culture and metagenomic methods [59] may lead to the identification of additional phage clusters, such as the novel methods reported by Serwer for the isolation of phage 0305phi8-36 [53].
(2) Transient host associations. Sampling methods relying on a single host genotype for phage discovery may preferentially detect phages that are specialist parasites of that host genotype. Phages Bastille, CP-51, and W.Ph. are reported to infect more than one species of *Bacillus* within the “ACT” family (*B. anthracis*, *B. cereus*, and *B. thuringiensis* [42]), and other work suggests host-switching may have contributed to the overall diversity of phages infecting *Bacillus* [12,60,61]. Host-switching or the presence of generalist parasites may lead to infrequent infections of a given host genotype, which are less likely to be sampled in culture.
(3) Variation in abundance. Spatial or temporal variation in phage composition has been shown for species communities [10,62]. Sampling schemes focused on specific geographic locations, ecosystems, seasons, or sampling over short time periods may increase sampling biases. For example, recently, three phages closely related to *Bacillus*-infecting phage SPP1 were identified, despite SPP1 being the sole representative of a cluster for 40 years [63].
(4) Relative abundance. Some phages may persist at high abundance relative to other phages within communities [64], decreasing the likelihood of sampling of low-abundance phage types in the presence of more common forms.
(5) Sporulation. Phage phi29 and CP-51 can persist following host sporulation, suppressing the lytic cycle [33,42,65]. The onset of sporulation following infection with a lytic phage could conceal the phage from traditional culture methods, as the phage may be unable to infect vegetative cells.

The proteomic analysis of YungSlug done here highlights that diverse phages continue to be discovered, even in bacterial host species that have previously been used to survey phage diversity. Here we also highlight multiple reasons why some genomically similar phages are sampled frequently (e.g., phages similar to Juglone, Bastille, and NotTheCreek), while others represent distinct or rarely sampled genotypes (e.g., SP-10, 0305phi8-36, and YungSlug). Together, this analysis of infrequently compared to frequently sampled clusters supports the notion that the discovery of rare clusters will continue to expand our knowledge of the genomic diversity of viruses. Furthermore, our work suggests homologs of such infrequently sampled clusters are not present in current metagenomic datasets, suggesting these sequences may represent a large pool of truly unexplored genetic diversity. While potential explanations include methodological and sampling biases, other explanations highlight potentially complex ecological interactions between bacterial species and their phage. Much work remains to be done to further our understanding of phages infecting the same host, as well as to reveal any universal patterns of evolutionary relationships.

## Data Availability

Raw genome sequence reads have been deposited in the NCBI Short Read Archive under accession SRR14661357. The assembled YungSlug genome has been deposited in the NCBI assembly database under ASM1326803. YungSlug genome annotation is posted under the accession number MT416612.2. Raw BLASTP search results and associated NCBI taxonomy strings have been archived in figshare under permanent object identifier 10.6084/m9.figshare.21554463.

## Supplementary Materials

The following supporting information can be downloaded at: https://www.mdpi.com/article/10.3390/v17091267/s1, Table S1: Proteins conserved in *Spounivirinae.*

## Abbreviations

The following abbreviations are used in this manuscript:

HHMI	Howard Hughes Medical Institute
SEA PHAGES	Science Education Alliance-Phage Hunters Advancing Genomics and Evolutionary Science
NCBI nr	National Center for Biotechnology Information, non-redundant protein records database
GCS	Gene content similarity
BLAST	Basic Local Alignment Search Tool
BLASTP	Standard protein Basic Local Alignment Search Tool
ACT family	*B. anthracis*, *B. cereus*, and *B. thuringiensis*
ICTV	International Committee on Taxonomy of Viruses
